# Predawn and high intensity application of supplemental blue light decreases the quantum yield of PSII and enhances the amount of phenolic acids, flavonoids, and pigments in *Lactuca sativa*

**DOI:** 10.3389/fpls.2015.00019

**Published:** 2015-02-26

**Authors:** Theoharis Ouzounis, Behnaz Razi Parjikolaei, Xavier Fretté, Eva Rosenqvist, Carl-Otto Ottosen

**Affiliations:** ^1^Department of Chemical Engineering, Biotechnology, and Environmental Technology, University of Southern DenmarkOdense, Denmark; ^2^Section for Crop Sciences, Department of Plant and Environmental Sciences, University of CopenhagenTaastrup, Denmark; ^3^Department of Food Science, Plant, Food & Climate, Aarhus UniversityAarslev, Denmark

**Keywords:** light-emitting diodes, blue light, stomatal conductance, secondary metabolites, chlorophyll fluorescence, lettuce, quantum efficiency of photosystem II

## Abstract

To evaluate the effect of blue light intensity and timing, two cultivars of lettuce [*Lactuca sativa* cv. “Batavia” (green) and cv. “Lollo Rossa” (red)] were grown in a greenhouse compartment in late winter under natural light and supplemental high pressure sodium (SON-T) lamps yielding 90 (±10) μmol m^−2^ s^−1^ for up to 20 h, but never between 17:00 and 21:00. The temperature in the greenhouse compartments was 22/11°C day/night, respectively. The five light-emitting diode (LED) light treatments were Control (no blue addition), 1B 06-08 (Blue light at 45 μmol m^−2^ s^−1^ from 06:00 to 08:00), 1B 21-08 (Blue light at 45 μmol m^−2^ s^−1^ from 21:00 to 08:00), 2B 17-19 (Blue at 80 μmol m^−2^ s^−1^ from 17:00 to 19:00), and 1B 17-19 (Blue at 45 μmol m^−2^ s^−1^ from 17:00 to 19:00). Total fresh and dry weight was not affected with additional blue light; however, plants treated with additional blue light were more compact. The stomatal conductance in the green lettuce cultivar was higher for all treatments with blue light compared to the Control. Photosynthetic yields measured with chlorophyll fluorescence showed different response between the cultivars; in red lettuce, the quantum yield of PSII decreased and the yield of non-photochemical quenching increased with increasing blue light, whereas in green lettuce no difference was observed. Quantification of secondary metabolites showed that all four treatments with additional blue light had higher amount of pigments, phenolic acids, and flavonoids compared to the Control. The effect was more prominent in red lettuce, highlighting that the results vary among treatments and compounds. Our results indicate that not only high light level triggers photoprotective heat dissipation in the plant, but also the specific spectral composition of the light itself at low intensities. However, these plant responses to light are cultivar dependent.

## Introduction

Light is one of the most significant variables affecting photosynthetic parameters and phytochemical concentrations in plants (Whitelam and Halliday, 2007; Kopsell and Kopsell, 2008). In the Northern European countries, supplementary light from high-pressure sodium (HPS) lamps is being used for up to 16 h per day (Paradiso et al., 2011). Light-emitting diodes (LEDs) represent a promising light source for greenhouses, which can be applied either as a main or supplementary light source (Marcelis et al., 2006). They have a variety of advantages compared to the traditional light systems, such as solid-state construction, low heat emission, longer lifetime, and higher energy conversion efficiency (Yorio et al., 2001; Morrow, 2008). They have been used as artificial light sources in closed plant production systems, where environmental conditions are controlled. They are available in narrow wavebands, making it possible to optimize light quality by choosing the right combination of wavelength (Morrow, 2008). A mixture of blue and red LEDs are commonly used as chlorophyll *a* (chl *a*) and *b* (chl *b*) mainly absorb in the blue and red region of the spectrum (Hopkins and Hüner, 2008; Son and Oh, 2013). Although red LEDs initially received great attention for use as a light source to drive photosynthesis, plants are evolutionary adapted to utilize a wide-spectrum of light (Briggs and Huala, 1999). Plants cannot develop optimally with red light alone, but need blue light as well to regulate other processes than photosynthesis and growth. Blue light has been reported to affect photomorphogenesis, vegetative growth, chlorophyll synthesis, stomatal opening, and secondary metabolism (Islam et al., 2012; Nascimento et al., 2013). Previous studies have reported that a minimal amount of blue light is necessary to achieve normal photosynthetic operation (Hogewoning et al., 2010; Trouwborst et al., 2010). However, the amount of blue light required for normal growth is species and/or cultivar dependent (Hogewoning et al., 2010; Johkan et al., 2010; Islam et al., 2012).

Lettuce is one of the most important leafy vegetables in greenhouse production and a well-studied crop for light quality responses (Dougher and Bugbee, 2001; Kim et al., 2004). The worldwide demand for lettuce is increasing because of its crispness, fresh appearance, as well as richness in phytochemicals (Llorach et al., 2008; Martínez-Sánchez et al., 2011). Secondary metabolites are essential phytochemicals that are affected by the light spectrum (Kopsell et al., 2004; Kopsell and Sams, 2013) and act as defense compounds as well as protectors from ultraviolet (UV) radiation and oxidants (Wink, 2010). Specifically, phenolic acids and flavonoids show antimicrobial, antioxidant, antifungal, and radical scavenging activities and act as blue and red pigments. Carotenoids have key roles as the major organic pigments found in chloroplasts and chromoplasts. All these secondary compounds protect photosynthetic organisms from harmful photooxidative processes or stress related events by increasing their amount (Lattanzio et al., 2006; Wink, 2010). In lettuce, it has been reported that blue LED lighting increased anthocyanins, xanthophylls, and β-carotene (Li and Kubota, 2009). In red leaf lettuce grown under blue LED lighting, an increase in anthocyanin, carotenoid, and chlorophyll content was reported (Johkan et al., 2010). Other researchers have also reported an increase in phenolic acids, flavonoids, and pigments in lettuce under blue LED lighting (Son and Oh, 2013), highlighting the importance of blue light in the production of such phytochemicals.

Chlorophyll fluorescence measurements have been proven to be a rapid, non-invasive, quantitative, and powerful method to assess the properties of the photosynthetic apparatus and to detect various stress effects and environmental changes (Baker and Rosenqvist, 2004). Not only can basic processes like electron transport rate (ETR) and heat dissipation through non-photochemical quenching be measured, but also the energy balance between yields of photochemistry (Φ_PSII_) and light regulated (Φ_NPQ_) and non-regulated (Φ_NO_) heat dissipation can also be measured (Maxwell and Johnson, 2000; Kramer et al., 2004). In addition to these approaches, stomatal regulation is also important for the photosynthetic status of the plant. Plant stomata respond to a variety of signals and stomatal opening is induced by many factors, such as CO_2_ concentration, air humidity and light intensity (Baroli et al., 2008). The photosynthetic apparatus is affected by both blue and red light (Saebø et al., 1995), while blue light acts as a signal for stomatal opening (Dougher and Bugbee, 1998; Shimazaki et al., 2007). Consequently, these approaches have been introduced to assess the fate of absorbed light (Kramer et al., 2004; Shimazaki et al., 2007) and concomitantly provide information for the physiological status of the plant.

It is difficult to perceive how lettuce responds to the production of secondary metabolites (SMs) after exposure to LED treatments because most existing studies compare different cultivars, different environmental and light conditions, and are focused on photosynthesis or crop yield, neglecting the important role of blue light on secondary metabolism (Dougher and Bugbee, 2001; Kim et al., 2004; Trouwborst et al., 2010; Islam et al., 2012). The functions of SMs have been under extensive research for their beneficial functions. Phenolic compounds demonstrate antioxidant, antimicrobial, antifungal, antitoxic, and radical scavenging properties (Kefeli et al., 2003; Lattanzio et al., 2006). Flavonoids constitute a group of phenolic compounds that affect the nutritional quality of plant-based food, such as lettuce and other leafy vegetables (Ebisawa et al., 2008; Hichri et al., 2011). With their functions, they help plants adapt to biotic and abiotic environmental changes or stressful events (Lynn and Chang, 1990). In our study, different blue LED light ratios and application timing were used to investigate the impact of supplemental blue light on lettuce growth, photosynthetic performance, and secondary metabolism. Although previous studies have focused on using combinations of red and blue LEDs, we selected only blue LED lighting in combination with SON-T as supplemental light source to explore the amount and timing/intensity of blue light needed to affect the content of pigments and phenolic compounds. In spite of the fact that red lighting has been proved to influence the photosynthetic ability, plant biomass, and plant growth (Kim et al., 2004), it was not applied together with blue lighting. The whole premise behind using pure blue LED light in combination with natural or supplemental light is based on our previous studies (Ouzounis et al., 2014a,b), which have shown that additional blue light increases the amount of SMs; however, those studies were performed almost solely with LEDs as supplemental light.

The objective of this study was to characterize the effect of blue light dose and timing on the physiological and morphological parameters as well as on the amount of pigments and phenolic compounds of *Lactuca sativa* “Batavia” and “Lollo Rossa” on a background of natural daylight. Different dose and timing applications could provide significant information, not only from a scientific point of view, but also for future lighting strategies since the LED technology is being implemented in greenhouses.

## Materials and methods

### Plant material and growth conditions

The experiment was conducted at University of Aarhus, Aarslev, Denmark (lat. 55.309° N, long. 10.439° E) and took place from January to March 2014 using small 4–5 leaf plants of green (*Lactuca sativa* “Batavia”) and red (*Lactuca sativa* “Lollo Rossa”) lettuce. The plants were sown in a commercial nursery; just after the first two-three permanent leaves developed they were transferred and conditioned in the experimental setting for a week before the experiment started. The temperature in the greenhouse compartments was set to 22°C and 11°C during the day and night, respectively (gradual shift within more than 2 h from 22 to 11°C at 17:00 till 08:00). The temperature was very close to the set points due to the outside cold temperatures. The main HPS supplementary light was SON-T lamps (400 W, HPS, Philips, Eindhoven, The Netherlands) yielding a photosynthetic photon flux density (PPFD) of 90 (±10) μmol m^−2^ s^−1^. These lamps were on from 21:00–08:00 to 12:00–17:00, the latter only if a set point of low light (7.5 W m^−2^ global radiation) was surpassed. The daily light integral during January and February was 6.1 mol m^−2^ d^−1^, while the natural light was less than 10% of the daily light integral. The experimental design was a randomized complete block design with sub blocks randomly assigned at the beginning of the experiment. Five LED light treatments were imposed using LED units solely in blue mode (FL300, Fionia Lighting, Søndersø, Denmark):

1. Control (daylight and SON-T).
2. Blue light at 45 μmol m^−2^ s^−1^ from 06:00 to 08:00 (1B 06-08).
3. Blue light at 45 μmol m^−2^ s^−1^ from 21:00 to 08:00 (1B 21-08).
4. Blue at 80 μmol m^−2^ s^−1^ from 17:00 to 19:00 (2B 17-19).
5. Blue at 45 μmol m^−2^ s^−1^ from 17:00 to 19:00 (1B 17-19).

**Figure 1:**
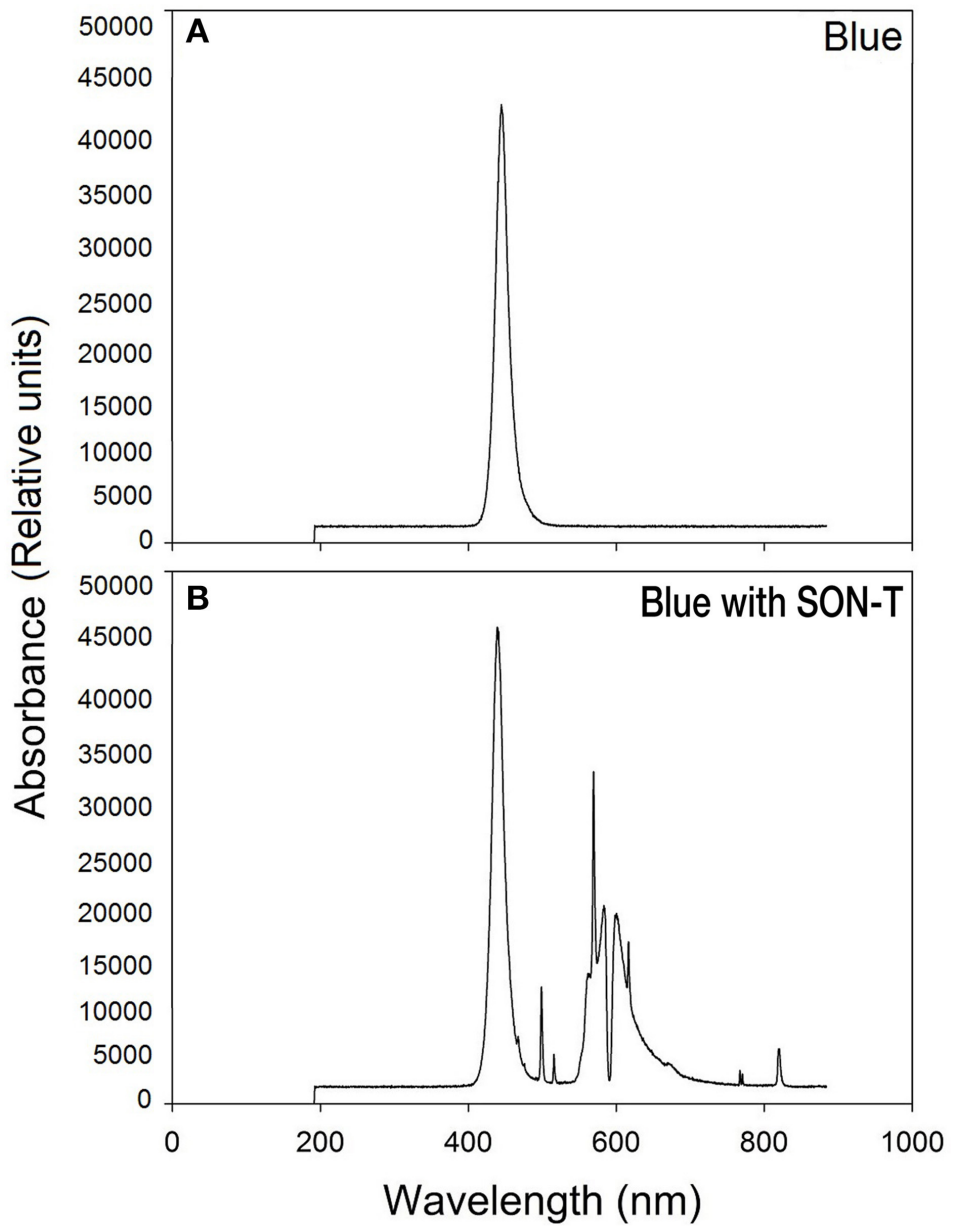
**Spectral distribution of blue (A) and blue with SON-T light (B)**.

The spectra for blue light *with* SON-T and blue light alone (measured without natural or supplemental lights) are shown in Figure 1. Spectra were recorded and averaged at three locations at plant height with a portable spectroradiometer (Jaz, Ocean Optics, Dunedin, USA).

### Physiological measurements

Plant growth was recorded at the end of the experiment (12 week old plants) and four green and four red fully developed lettuce plants per treatment were harvested for fresh weight (FW) and dry weight (DW) measurements. Fresh weight was determined using a PG5002-S DeltaRange® scale (Mettler-Toledo A/S, Glostrup, Denmark). Dry weight was determined after 24 h at 70°C with a dry heat oven (Holm and Halby A/S, Brøndby, Denmark). Stomatal conductance (g_s_) was measured with the SC-1 leaf porometer (Decagon Devices Inc., Pullman, Washington, USA) using four different leaves from different plants per treatment and cultivar (leaves selected from the outer layer and were fully developed). The porometer was calibrated and fresh desiccant was used each time before measurements to ensure accurate conductance measurements. The measurements were taken between 09:00 and 14:00 to assure fully active plants, outside the time periods when blue light was applied in any of the treatments. Every effort was made to maintain thermal equilibrium in the sensor head. Four different leaves from different plants per treatment and cultivar were chosen randomly for measuring the maximum photochemical efficiency of PSII (F_v_/F_m_) with a MINI-PAM (Walz, Effeltrich, Germany). Three different fully developed leaves from different plants per treatment from both green (“Batavia”) and red (“Lollo Rossa”) leaf lettuce were taken randomly for measuring the quantum efficiency of PSII (Φ_PSII_), the non-photochemical quenching (NPQ), the quantum yield of the down-regulatory non-photochemical quenching (Φ_NPQ_), the quantum yield of other non-photochemical losses (Φ_NO_), the fraction of open PSII centers (q_L_), and the electron transport rate (ETR) under 100, 500, and 1100 μmol m^−2^ s^−1^ using the portable fluorometer PAM 2500 (Walz, Effeltrich, Germany) operated by PAMWin-3 software. The sum of yields for the dissipative processes for the energy absorbed by PSII is unity: Φ_PSII_ + Φ_NPQ_ + Φ_NO_ = 1 (Kramer et al., 2004). All leaf samples were dark adapted for at least 20 min before measurement.

### Phenolic compounds

Four different leaves from different plants per treatment (plant material amalgamated) from both green and red leaf lettuce cultivars were collected and stored at −80°C for later analysis by HPLC. Approximately 3 (±0.2) g were ground with liquid nitrogen and 10 mL of 80% methanol (MeOH, VWR International, Herlev, Denmark) was used for extraction. All samples were extracted in darkness for at least 90 min and 1 mL was filtered on 0.2 μM micro filters (Whatman GmbH, Dassel, Germany) and put into HPLC vials; all samples were kept at −80°C until later analysis with HPLC (Shetty et al., 2011). Extracts were analyzed by a Dionex 3000 Ultimate HPLC-PDA on a Dionex-Chromeleon Chromatography Data System (Thermo Scientific™ Dionex™ Chromeleon™ 7.1 Chromatography Data System, Sunnyvale, CA, USA). Separations were performed on a Zorbax Eclipse XDB-C18 column (150 × 4.6 mm, 5 μm; Agilent, Santa Clara, CA, USA) with the following solvents: solvent A = 0.1 % formic acid (HPLC grade, purity of 99%; Sigma-Aldrich, Denmark A/S, Copenhagen, Denmark) in water and solvent B = 0.1 % formic acid in acetonitrile (HPLC grade; Fisher Scientific, Waltham, MA, USA). The column was maintained at 30°C using a thermostated column compartment. The HPLC method had a run time of 66-min and the flow was 1.0 mL min^−1^. The solvent gradient was from 0 to 5 min isocratic 1% B, from 5 to 45 min linear gradient from 1 to 100% B, from 45 to 55 min isocratic 100% B, from 55 to 60 min linear gradient from 100 to 1% B, and from 60 to 66 min isocratic 1% B. Eluted compounds came from a 10-μL injection. The phenolic acids and flavonoids were monitored at 320 and 360 nm, respectively, and UV spectra were recorded from 210 to 600 nm. Analysis of selected representative extracts was also performed under the same HPLC conditions with an HPLC hyphenated with a mass spectrometer (Accela LTQ ion trap XL ETD, Thermo Scientific™, Waltham, MA, USA), making comparison of HPLC-DAD and LC-MS chromatograms and spectra completely reliable, and thus allowing identification of metabolites. The software used for interpreting the results of the LC-MSMS was Xcalibur version 2.0.7. Flavonoids and phenolic acids were quantified as equivalents of rutin and chlorogenic acid (Sigma-Aldrich A/S, Copenhagen, Denmark), respectively in extracts from external calibration curves. Every effort was made to reduce any effects of light and/or thermal degradation of leaf tissue pigments during extraction and HPLC analysis. Extraction of leaf tissue pigments was performed in dim light and at room temperature (20°C). Extracts were then transferred in brown HPLC vials and placed in the HPLC sampler, whose temperature was set to 5°C. Samples were also filtered into amber HPLC vials that reduce transmitted light and protected when run on the HPLC by a tinted shield covering the auto-sampler.

### Pigments

Four different leaves from different plants per treatment (plant material amalgamated) from both green and red leaf lettuce were taken randomly and stored at −80°C for later analysis by HPLC. Approximately 3 (±0.2) g of plant material were ground with liquid nitrogen and 80% acetone was used for extraction. Samples were extracted in darkness for at least 90 min and were put in vials and kept at −80°C until later analysis with HPLC (Shetty et al., 2011). Separations were performed on a Zorbax Eclipse XDB-C30 column (5 μm, 150 × 4.6 mm; Agilent, Hørsholm, Denmark) with the following solvents: solvent A = 100% methanol (HPLC grade, Sigma-Aldrich) and solvent B = 50% hexane in 50% isopropanol (HPLC grade; Fisher Scientific). The column was maintained at 30°C using a thermostated column compartment. The flow was 1.0 mL min^−1^ with a run time of 30-min followed by a 10-min equilibration before the next injection. The mobile phase was run at the following gradient: 0 min, 0% B; 14 min, 75% B; 20 min, 100% B; 22 min, 0% B. Eluted compounds came from a 20-μL injection. Pigments were quantified in extracts by HPLC-DAD on a Dionex-Chromeleon Chromatography Data System. Chromatographic conditions were based on the lab-established HPLC method. The pigments were monitored at 450 nm, respectively, and UV spectra were recorded from 210 to 600 nm. Separations were performed under the same HPLC conditions as used for LC-MS analyses, thus making comparison of chromatograms and spectra completely reliable. The software used for interpreting the results of the LC-MSMS was Xcalibur version 2.0.7. Carotenoids were determined in extracts from external calibration curves of β-carotene. Every effort was made to reduce any effects of light and/or thermal degradation of leaf tissue pigments during extraction and HPLC analysis. Samples were also filtered into amber HPLC vials that reduce light and protected when run on the HPLC by a tinted shield covering the autosampler.

Four different leaves from different plants per treatment (plant material amalgamated) from both green and red leaf lettuce were taken randomly and stored at −80°C for later chlorophyll analysis. The chlorophyll content was measured by spectrophotometry using a DR 3900 spectrophotometer (Hach Lange Aps, Brønshøj, Denmark). The optical density was measured at 663 nm for chlorophyll *a* (chl *a*) and at 646 nm for chlorophyll *b* (chl *b*). The concentrations of chl *a* and chl *b* were determined from the following equations (Harborne, 1984):

Chlorophyll a (mg l^−1^) = 12.21A_663_−2.81A_646_
Chlorophyll b (mg l^−1^) = 20.13A_646_−5.03A_663_

Statistical analysis was carried out using the R-language “stat” package (R Core Team, 2013). Analysis of variance was done with treatment as random factors. Least significance difference (*P* < 0.05) was determined according to LSD test.

## Results

### Plant growth and development

**Figure 2:**
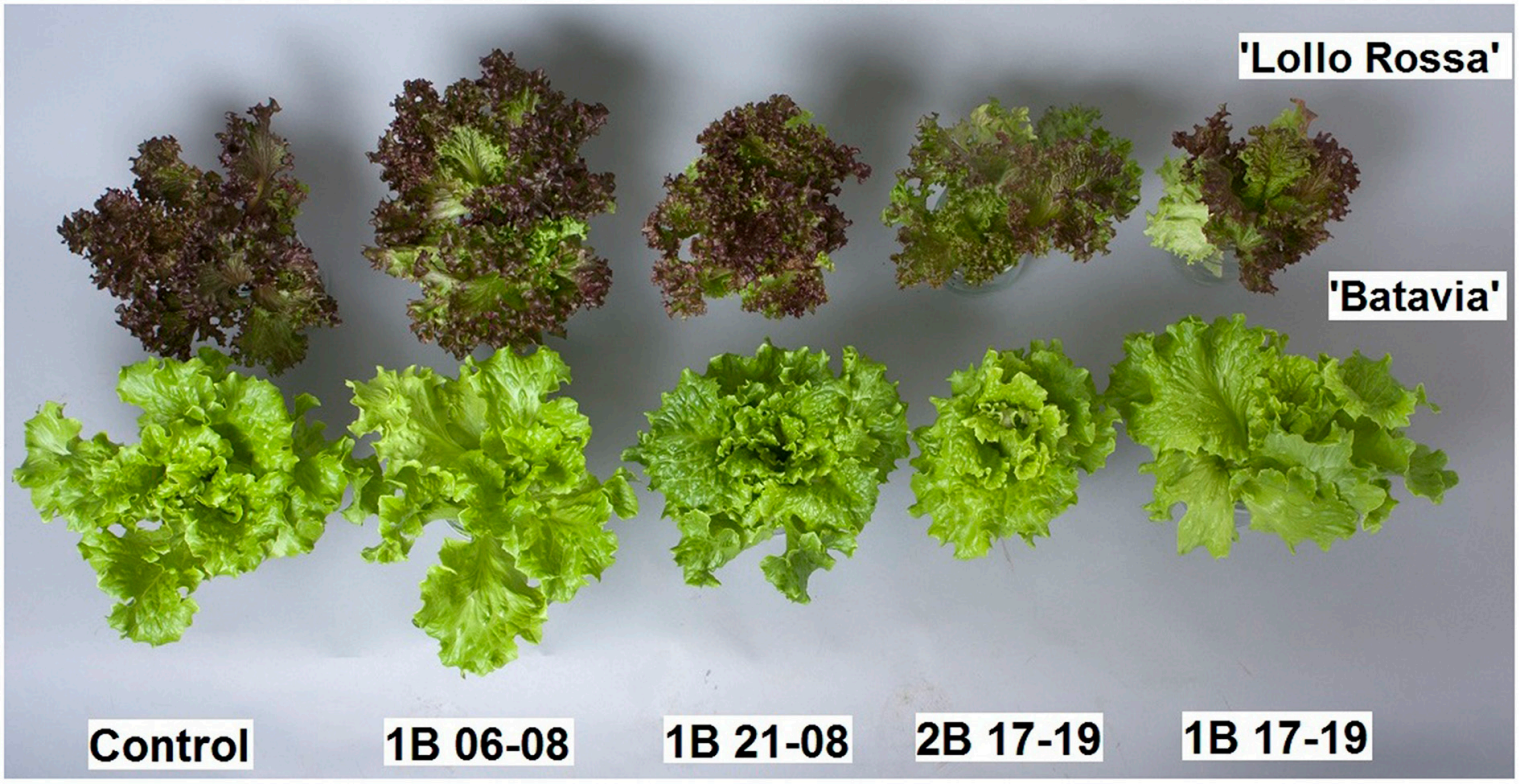
**Morphological differences of *Lactuca sativa* “Batavia” and “Lollo Rossa” grown under the five different LED treatments: Control (no blue addition), Blue light at 45 μmol m^−2^ s^−1^ from 06:00 to 08:00 (1B 06-08), Blue light at 45 μmol m^−2^ s^−1^ from 21:00 to 08:00 (1B 21-08), Blue at 80 μmol m^−2^ s^−1^ from 17:00 to 19:00 (2B 17-19), Blue at 45 μmol m^−2^ s^−1^ from 17:00 to 19:00 (1B 17-19)**.

Fresh weight (FW) as well as dry weight (DW) were not affected in any of the cultivars and no statistical difference was observed among the treatments (data not shown). Plants grown under 1B 06-08 had comparable leaf expansion with the Control for both “Batavia” and “Lollo Rossa,” while plants grown under 1B 21-08 and 2B 17-19 were clearly more compact (Figure 2). 1B 17-19 showed smaller leaf area in “Lollo Rossa,” but not in “Batavia” (visual observations). Due to the extreme curliness of the leaves it was not possible to measure the leaf area without damages.

### Stomatal conductance (g_s_) and chlorophyll fluorescence parameters

**Figure 3:**
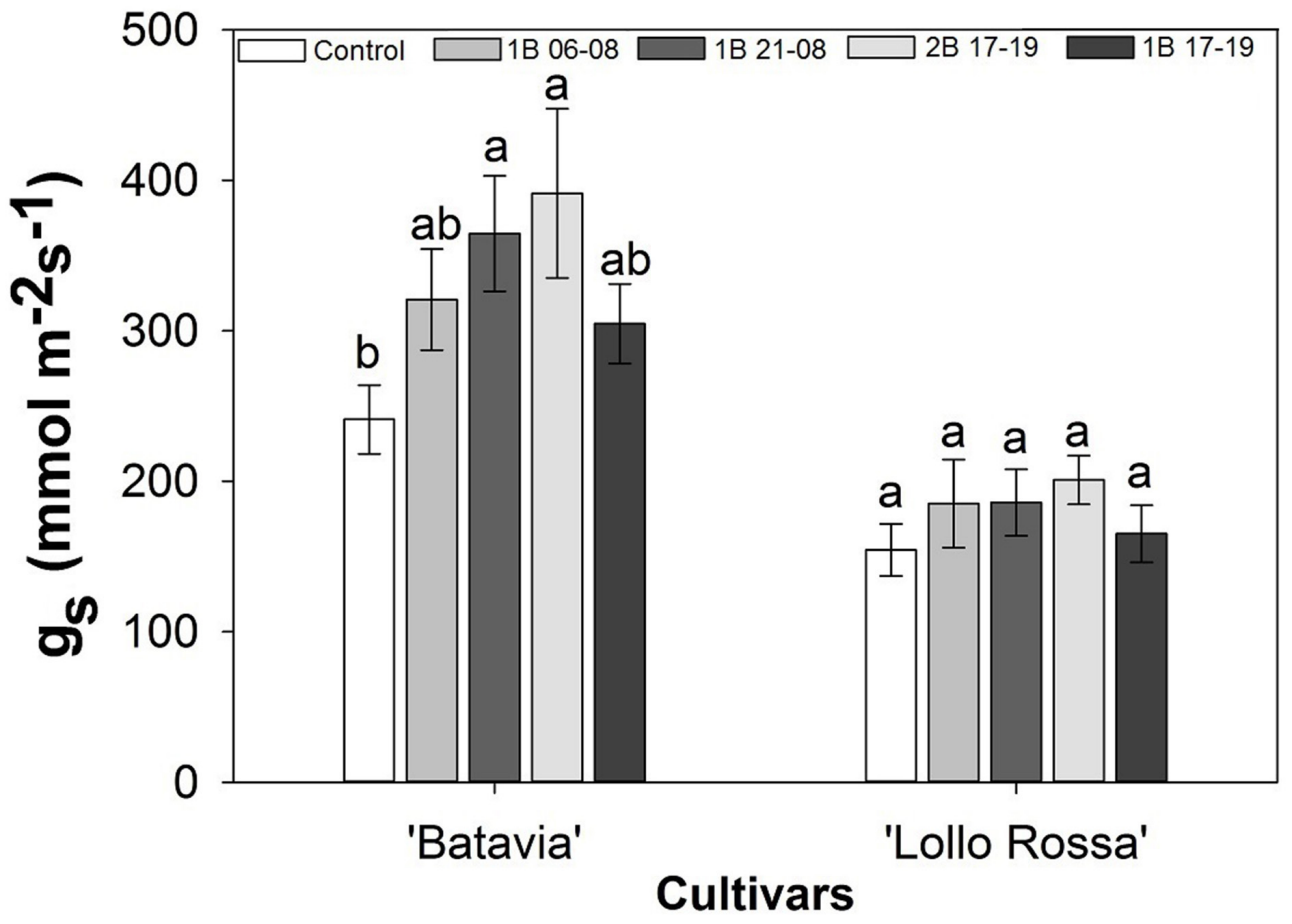
**Stomatal conductance (g_s_) of *Lactuca sativa* “Batavia” and “Lollo Rossa” grown under the five different LED treatments with the same abbreviations as in Figure 2**. Data are mean values (*n* = 20, five replications, four different leaves from different plants per treatment and cultivar) ± SE. Assignment of the same letters indicates values that are not significantly different at *P*-values < 0.05 within treatments.

Stomatal conductance was affected by the blue light treatments (Figure 3). In “Batavia,” 2B 17-19 and 1B 21-08 exhibited higher g_s_ than the Control with 1B 06-08 and 1B 17-19 showing intermediate values. In “Lollo Rossa” a similar pattern was found between the treatments but the differences were too small to be significant.

**Figure 4:**
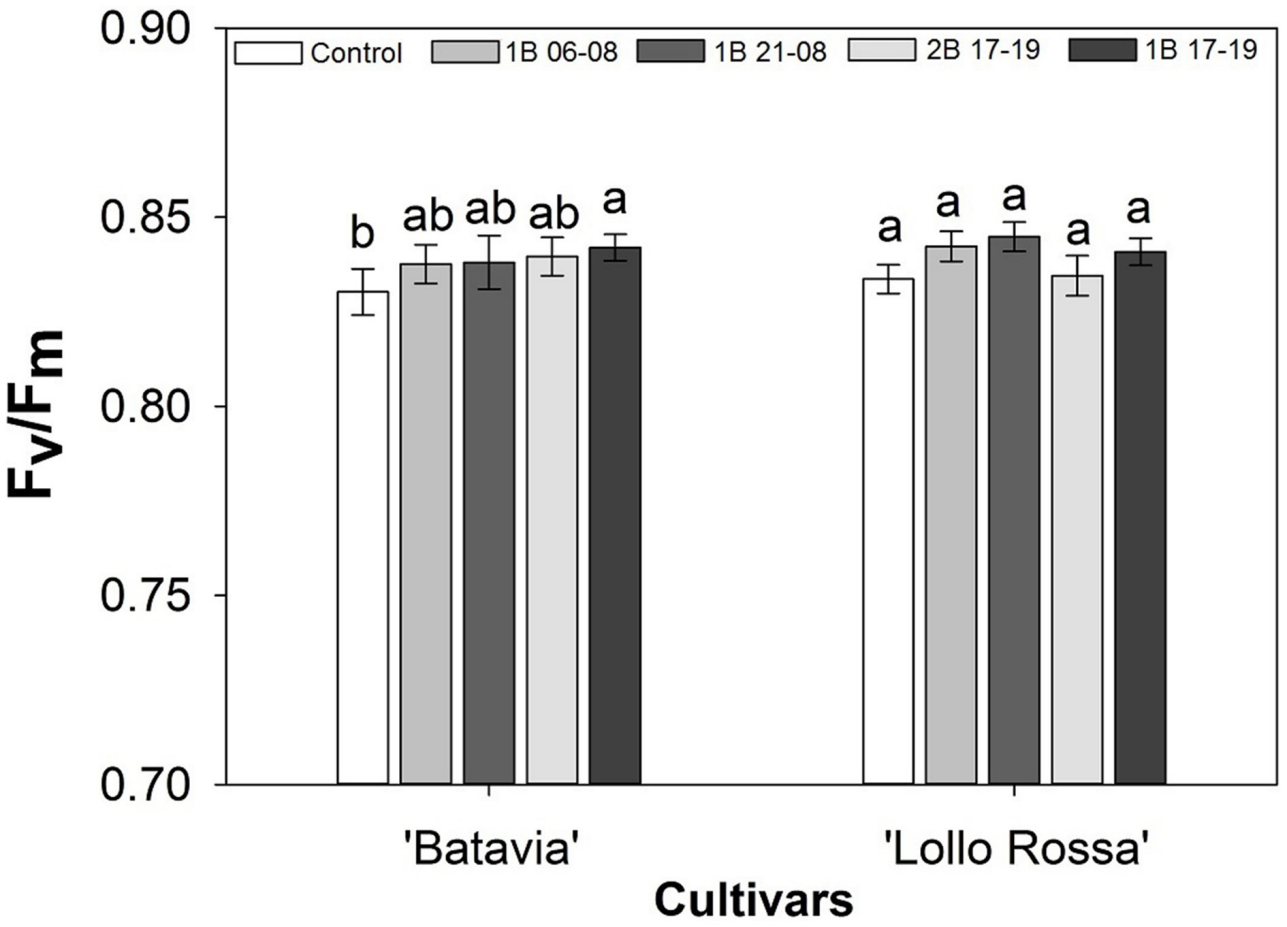
**Maximum photochemical efficiency of PSII (F_v_/F_m_) of *Lactuca sativa* “Batavia” and “Lollo Rossa” grown under the five different LED treatments with the same abbreviations as in Figure 2**. Data are mean values (*n* = 16, four replications, four different leaves from different plants per treatment and cultivar) ± SE. Assignment of the same letters indicates values that are not significantly different at *P*-values < 0.05 within treatments.

The average F_v_/F_m_ was not affected by the light treatments (Figure 4). In “Batavia,” 1B 17-19 was significantly higher than the Control with 2B 17-19, 1B 21-08, and 1B 06-08 showing intermediate values. The difference between treatments was non-significant in “Lollo Rossa.”

**Figure 5:**
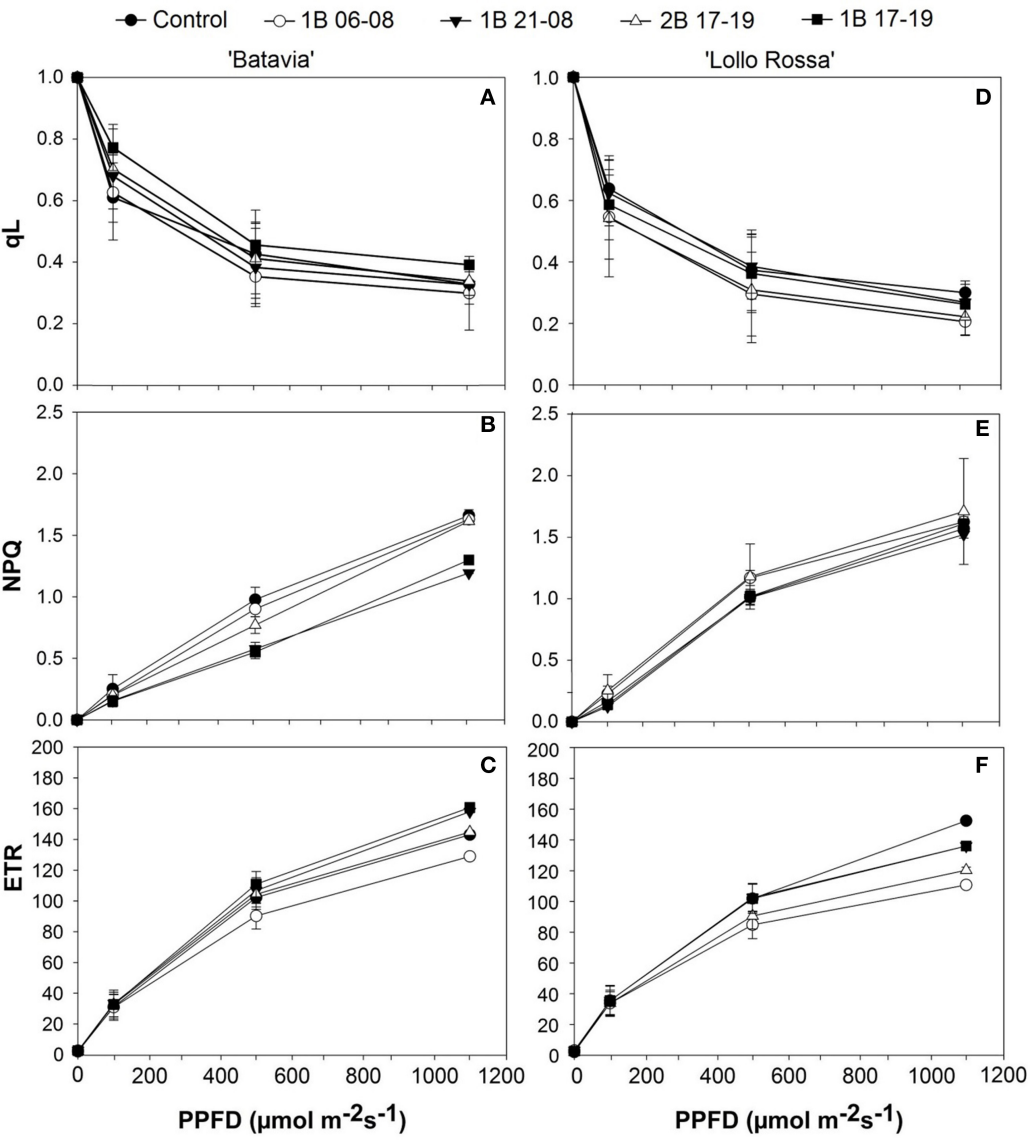
**Fraction of open PSII centers (q_L_), non-photochemical quenching (NPQ), and electron transport rate (ETR) of *Lactuca sativa* “Batavia” **(A–C)** and “Lollo Rossa” **(D–F)** grown under the five different LED treatments with the same abbreviations as in Figure 2**. Data are mean values (*n* = 3, three different leaves from different plants per treatment and cultivar) ± SE.

Parameters such as q_L_, NPQ, and ETR were also calculated (Figure 5). The ETR was highest for 1B 21-08 and 1B 17-19 in “Batavia” (Figure 5C), followed by the 2B 17–19, the Control, and 1B 06-08 at 500 and 1100 μmol m^−2^ s^−1^. In “Lollo Rossa,” ETR was highest (significant) for the Control, followed by 1B 21-08, 1B 17-191B 17-19, 2B 17-19, and 1B 06-08 at 500 and 1100 μmol m^−2^ s^−1^. In “Batavia,” the NPQ (Figure 5B) showed higher steep and values for Control, 1B 06-08, and 2B 17-19 while 1B 21-08 and 1B 17-19 had lower values at 500 and 1100 μmol m^−2^ s^−1^. In “Lollo Rossa,” the NPQ was similar among all treatments (Figure 5E). The q_L_ was similar for “Batavia” at all light levels (Figure 5A) with non-significant differences among the treatments. A similar situation was observed for “Lollo Rossa” (Figure 5D).

**Figure 6:**
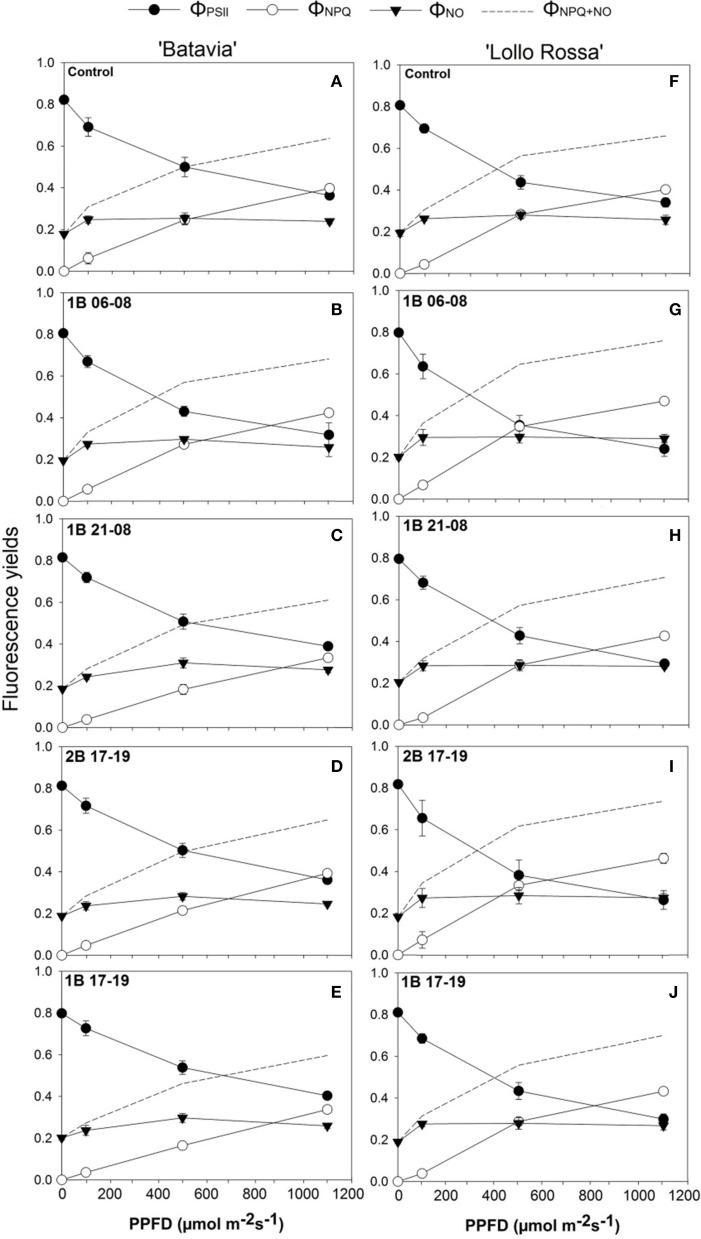
**Quantum efficiency of PSII (Φ_PSII_), yield for dissipation for down-regulation (Φ_NPQ_), and yield of other non-photochemical losses (Φ_NO_) of *Lactuca sativa* “Batavia” (A–E) and “Lollo Rossa” (F–J) grown under the five different LED treatments with the same abbreviations as in Figure 2**. Data are mean values (*n* = 3, three different leaves from different plants per treatment and cultivar) ± SE.

The yields of Φ_PSII_, Φ_NPQ_, and Φ_NO_ are presented in Figure 6. Compared to the Control, only 1B 06-08 (Figure 6B) demonstrates a slight shift from Φ_PSII_ to heat dissipation at a lower PPFD level. The Φ_PSII_ line intersects with Φ_NPQ_ at around 900 μmol m^−2^ s^−1^ in comparison with the Control (Figure 6A) where the intersection occurs at a higher level at around 1000 μmol m^−2^ s^−1^. The rest of the treatments for the “Batavia” (1B 21-08, 2B 17-19, and 1B 17-19) showed a similar shift. On the other hand, in “Lollo Rossa,” a trend expressed as shift from Φ_PSII_ to heat dissipation at lower PPFDs was observed in all the treatments compared to the Control. The Φ_PSII_ andΦ_NPQ_ lines intersected at around 500, 800, 700, and 800 μmol m^−2^ s^−1^, for 1B 06-08, 1B 21-08, 2B 17-19, and 1B 17-19, respectively; for the Control the intersection occurred at a higher level of PPFD (around 950 μmol m^−2^ s^−1^). 1B 06-08 (Figure 6G) showed the greatest shift to heat dissipation, followed by 2B 17-19 (Figure 6I), 1B 17-19 (Figure 6J), and 1B 21-08 (Figure 6H). The Φ_NO_ was similar for all treatments including the Control.

### Phenolic acids, flavonoids, and pigments

**Figure 7:**
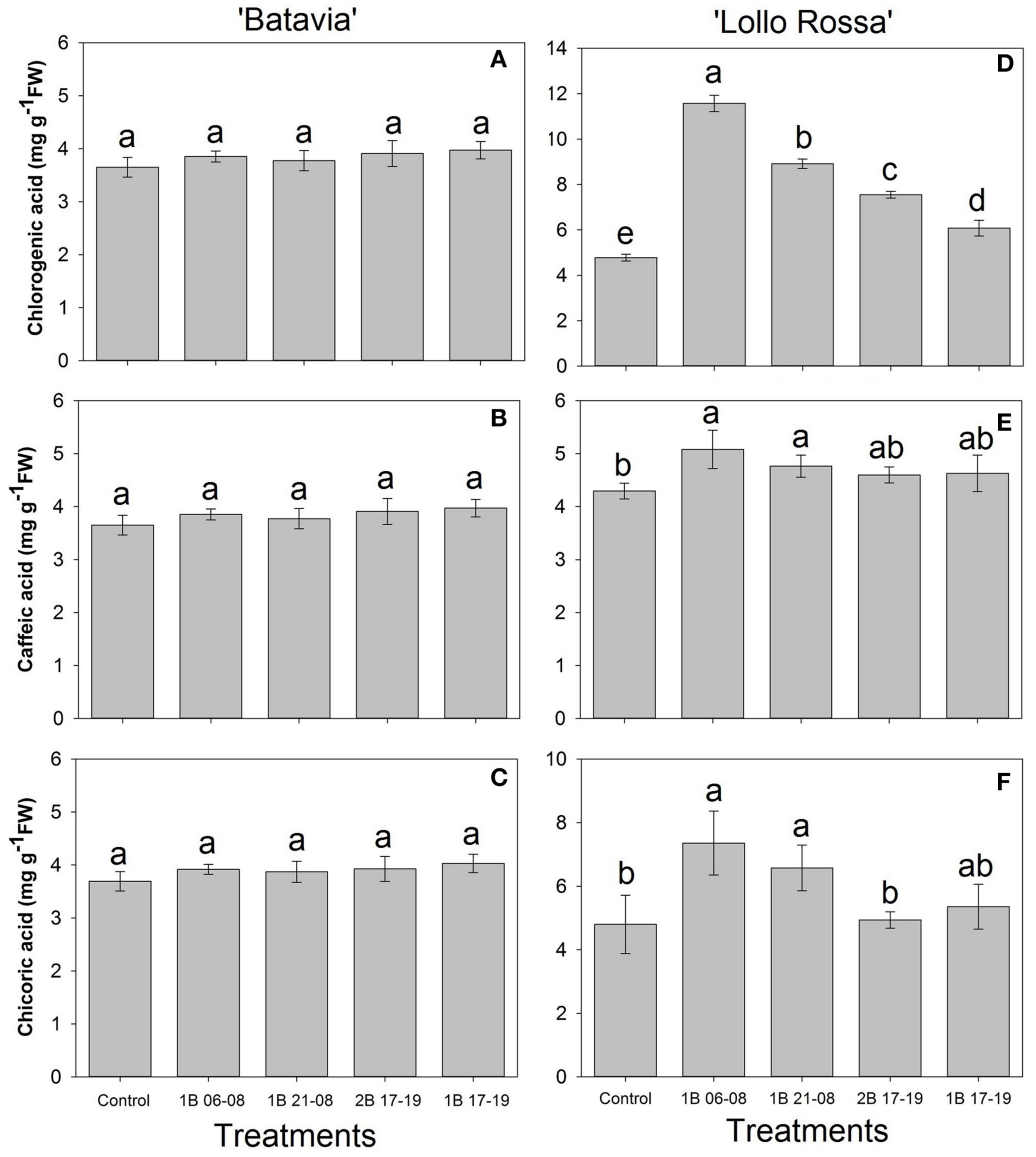
**Phenolic acid (chlorogenic acid, caffeic acid, and chicoric acid) of *Lactuca sativa* “Batavia” (A–C) and “Lollo Rossa” (D–F) grown under the five different LED treatments with the same abbreviations as in Figure 2**. Data are mean values (*n* = 4, four different leaves from different plants per treatment and cultivar) ± SE. Assignment of the same letters indicates values that are not significantly different at *P*-values < 0.05 within phenolic acids and treatments.

For “Batavia” (Figures 7A–C), the amount of phenolic acids was unaffected by the treatments. On the contrary, in “Lollo Rossa” (Figures 7D–F), the same phenolic acids were significantly higher after enrichment by blue light. For chlorogenic acid, 1B 06-08, 1B 21-08, 2B 17-19, and 1B 17-19 were all significantly different in descending order from the Control. For caffeic acid, only 1B 06-08 and 1B 21-08 were significantly higher than the Control. For chicoric acid, 1B 06-08 and 1B 21-08 showed the highest amount, followed by 1B 17-19, 2B 17-19, and the Control. 1B 06-08 and 1B 21-08 were significantly different from the Control.

**Figure 8:**
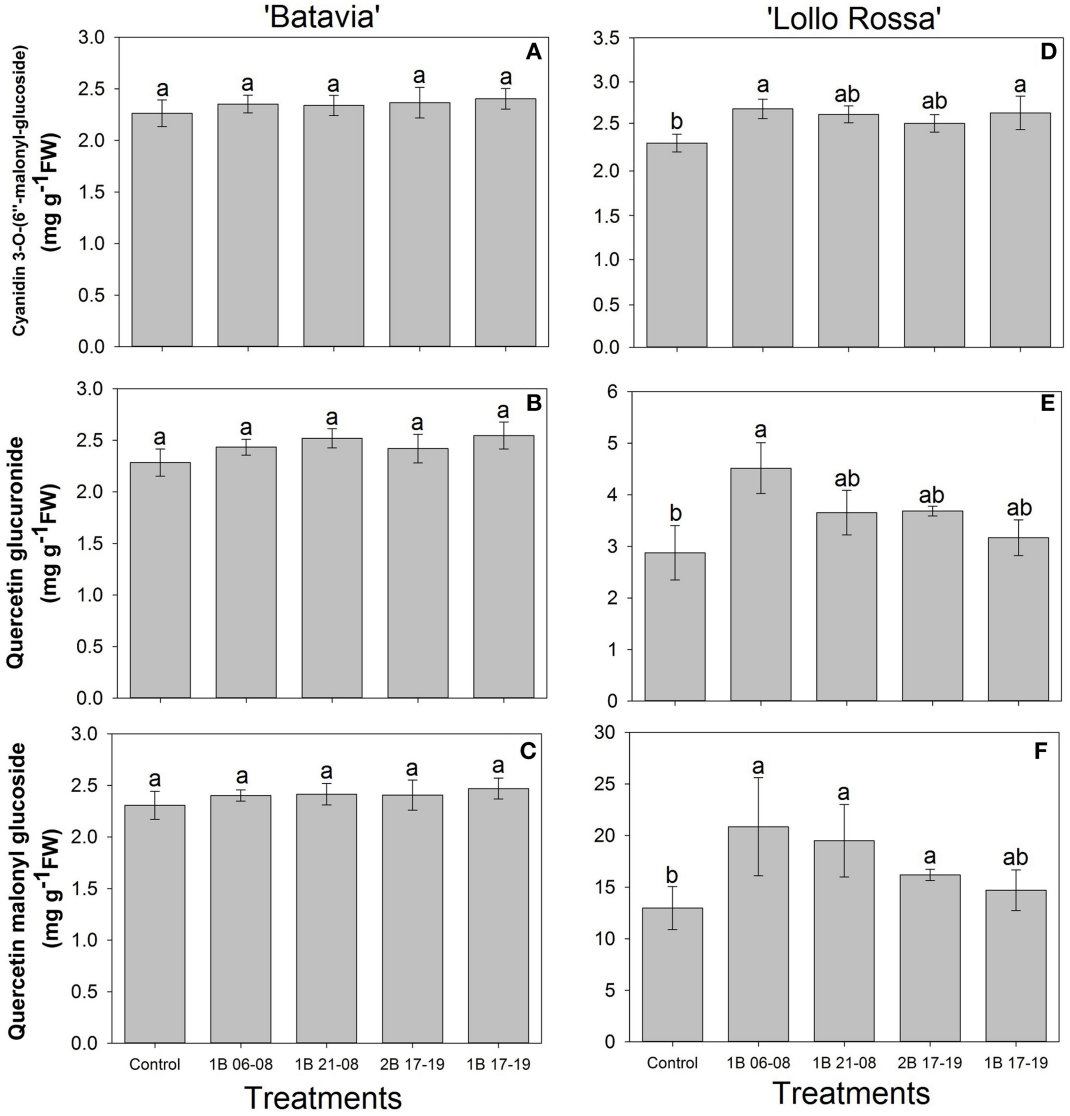
**Flavonoid content [cyanidin 3-O-(6″-malonyl-glucoside), quercetin glucuronide, quercetin malonyl glucoside] of *Lactuca sativa* “Batavia” (A–C) and “Lollo Rossa” (D–F) grown under the five different LED treatments with the same abbreviations as in Figure 2**. Data are mean values (*n* = 4, four different leaves from different plants per treatment and cultivar) ± SE. Assignment of the same letters indicates values that are not significantly different at *P*-values < 0.05 within flavonoids and treatments.

The amount of flavonoids showed similar responses as the phenolic acids with no significant difference in “Batavia” (Figures 8A–C). In “Lollo Rossa” (Figures 8D–F), cyanidin 3-O-(6″-malonyl-glucoside) was significantly higher than the Control in 1B 06-08 and 1B 17-19 with 1B 21-08 and 2B 17-19 having intermediate values. Quercetin glucuronide and quercetin malonyl glucoside showed the same response pattern as chlorogenic acid but with smaller and less significant differences. Only 1B 06-08 had significantly higher concentration of quercetin glucuronide than the Control. There was no significant difference in the concentration of quercetin malonyl glucoside in the treatments enriched with blue light but 1B 06-08, 1B 21-08, and 2B 17-19 were significantly higher than the Control.

**Figure 9:**
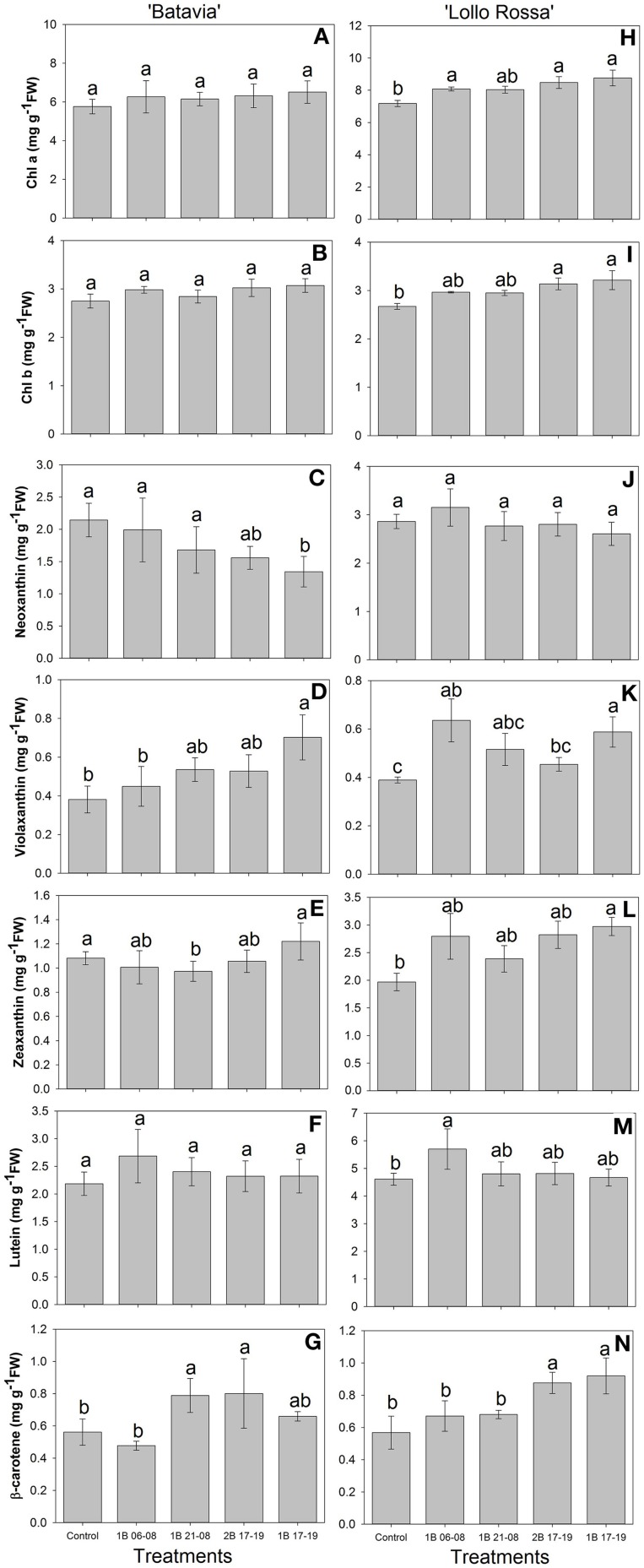
**Pigment content (chl *a*, chl *b*, neoxanthin, violaxanthin, zeaxanthin, lutein, β-carotene) of *Lactuca sativa* “Batavia” (A–G) and “Lollo Rossa” (H–N) grown under the five different LED treatments with the same abbreviations as in Figure 2**. Data are mean values (*n* = 4, four different leaves from different plants per treatment and cultivar) ± SE. Assignment of the same letters indicates values that are not significantly different at *P*-values < 0.05 within pigments and treatments.

The content of the pigments demonstrated a similar trend with the amount of phenolic acids and flavonoids for “Batavia” (Figure 9). Predominantly, no significant effect was shown in the high-concentration pigments in “Batavia,” however, some significant differences were observed in “Lollo Rossa” (Figures 9H–N). The pigment content showed a similar trend for “Batavia” (Figures 9A–G). In the latter, chl *a* and *b* increased in the treatments with blue light. Both chl *a* and *b* were highest for 1B 17-19, followed by 2B 17-19, 1B 06-08, 1B 21-08, and the Control. 1B 17-19 and 2B 17-19 were significantly different from the Control. Violaxanthin, zeaxanthin, lutein, and β-carotene exhibited higher amount when compared to the Control, with most of the blue light treatments being also statistically significant. Additionally, neither in “Batavia” nor in “Lollo Rossa” were there significant differences found with respect to chl *a:b* ratio (data not shown).

**Figure 10:**
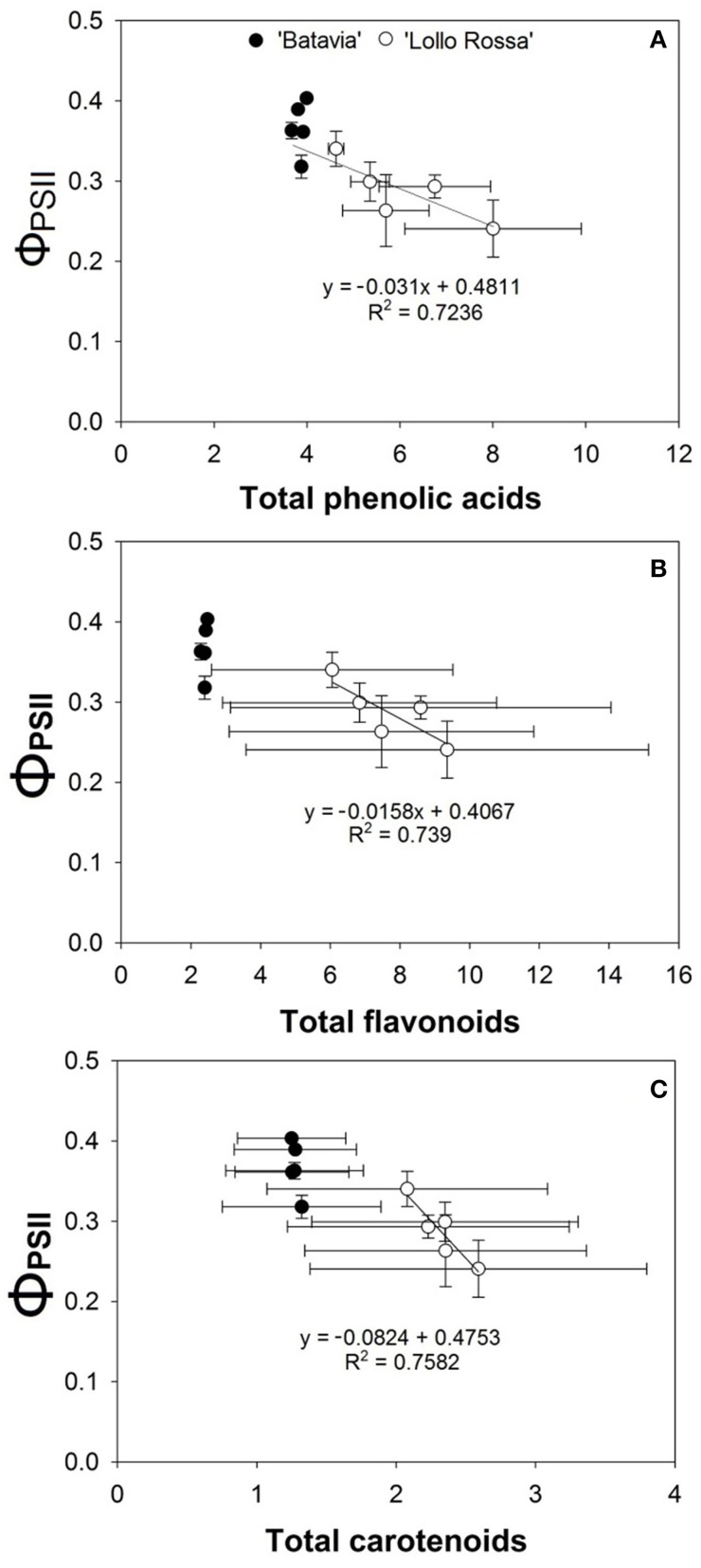
**Correlation between the quantum efficiency of PSII (Φ_PSII_) at 1100 μ mol m^−2^ s^−1^ and the amount of (A) phenolic acids, (B) flavonoids, and (C) carotenoids of *Lactuca sativa* “Batavia”(•) and “Lollo Rossa” (°) grown under the five different LED treatments with the same abbreviations as in Figure 2**. Data are mean values (*n* = 3, three different leaves from different plants per treatment and cultivar) ± SE.

The correlation between the respective metabolite group and the quantum efficiency of PSII (Φ_PSII_) are shown in Figure 10. While “Batavia” did not show any correlation, “Lollo Rossa” showed a positive correlation between Φ_PSII_ and each of the secondary metabolite category, i.e. phenolic acids (Figure 10A), flavonoids (Figure 10B), and carotenoids (Figure 10C).

## Discussion

### Plant growth and development

Plant biomass was not affected by the blue light treatments in any of the cultivars confirming earlier results on lettuce and other species (Dougher and Bugbee, 2001; Yorio et al., 2001). This should not come as a surprise as red light is the primary light affecting stem elongation, biomass production, and photomorphogenic responses via the phytochrome photoreceptor (Sager and McFarlane, 1997). All the plants under higher blue light ratio had a more compact appearance with no noticeable morphological abnormalities. Specifically, besides the given cultivar specific marginal curling on the leaf edges, no leaf blade curling was observed under blue light as previously found under monochromatic red supplementary light in pot roses (Ouzounis et al., 2014a). Blue light is perceived by the phototropins phot1 and phot2, which are responsible for regulating leaf flattening by suppressing the leaf curling activity of phytochrome B (Kozuka et al., 2013). Additionally, in lettuce, exposure to blue light could reverse morphological abnormalities and sustain a normal plant growth (Johkan et al., 2010). Our results indicate that lettuce grown under blue LED lighting did not enhance FW and DW, but rather partitioned assimilates for other processes, possibly leaf thickening or the production of SMs and carbohydrates.

Light is the major regulator of g_s_, since stomata should provide the leaf with the CO_2_ needed for net photosynthesis. Red light seems to be the signal (sensed via the internal CO_2_ concentration) which decreases when net photosynthesis is high and therefore the need for CO_2_ is high (Shimazaki et al., 2007). High light intensity has been reported to increase g_s_ in silver birch (Sellin et al., 2008). Increased amount of blue light clearly increased g_s_, especially in green lettuce (Figure 3). The treatment with the highest blue light intensity (2B 17-19) was the one that had the highest value of g_s_ in both cultivars, though the effect was more prominent in the green “Batavia.” It seems that high blue light intensity increased g_s_ in general and the fact that the measurements were taken between 09:00 and 14:00 indicates a remaining effect from the blue LED treatments even after blue LED light was switched off. Supplying light at 100 μmol m^−2^ s^−1^ for 16 h per day in cucumbers is sufficient to grow normal plants under different light spectra or blue and red LED light combinations (Hogewoning et al., 2010; Savvides et al., 2012). The current observations are possibly attributed to the involvement of phototropins and cryptochromes (blue light photoreceptors) in the regulation of g_s_ (Whitelam and Halliday, 2007; Hogewoning et al., 2010). Several other studies have also shown that blue light increases g_s_ possibly through the phototropins, cryptochromes, or the carotenoid zeaxanthin (Kinoshita et al., 2001; Mao et al., 2005). Additionally, it is worth mentioning that the apparent increase in g_s_ could be accredited to a synergistic or additive effect of several stomatal traits, such as density, length, width, pore length, or pore aperture (Boccalandro et al., 2012; Savvides et al., 2012).

### Chlorophyll fluorescence

The F_v_/F_m_ parameter measures the intrinsic efficiency of PSII under normal conditions with an optimal value of 0.83 for most healthy plant species (Björkman and Demmig, 1987). A lower F_v_/F_m_ value could be used as an indication of the stress level of plants, demonstrating possible photoinhibition under stressful events (Maxwell and Johnson, 2000; Baker and Rosenqvist, 2004). In our study, we observed values close to the optimal value when plants were untreated (under supplementary or natural light) or grown under blue LED lighting (Figure 4). There was a slightly lower value for the Control of “Batavia” in comparison with the other treatments, but since the F_v_/F_m_ value was close to the generally accepted maximum value of 0.83 (Björkman and Demmig, 1987), it indicated no significant plant stress.

In the red lettuce, ETR was lower for the blue LED treatments compared to the Control values. Specifically, these observations were more prominent in the predawn (1B 06-08) or the double intensity (2B 17-19) applications (Figure 5F), indicating that application timing and intensity is important for the ETR of red lettuce. It is also worth noting that we performed the measurements with the internal light source of a fluorometer, hence our results show a remaining effect of the blue light applications on the plants after the different treatments and this was expressed with lower ETR and Φ_PSII_ values (Figures 5, 6). The reduction of the Φ_PSII_ under blue light could be attributed to changes in the energy distribution between photosystems (Evans, 1986). It has been reported that ETR increased for *Platanus orientalis* with increasing blue LED lighting (40 and 80%), but decreased for *Zea mays*, demonstrating that the effect could also be cultivar or species dependent (Loreto et al., 2009). Indeed, we have also shown that two different cultivars of *Phalaenopsis* responded differently to varying amounts of blue light when we measured the same parameters (Ouzounis et al., 2014b).

The amount and timing of blue light applied seems to have an impact on Φ_PSII_ with a different effect on “Batavia” and “Lollo Rossa” lettuce. The shift from Φ_PSII_ to heat dissipation depending on the application timing and intensity was not significant in the green lettuce. In other words, the intersection points of Φ_PSII_ and Φ_NPQ_ were around 900 μmol m^−2^ s^−1^ or above (Figure 6). Specifically for 1B 21-08 and 1B 17-19, no intersection was observed even at 1200 μmol m^−2^ s^−1^. Only 1B 06-08 showed a steeper Φ_PSII_ and more gradual Φ_NPQ_ (Figure 6B). 1B 06-08 was the only treatment with additional blue light that was applied in the morning from 06:00 to 08:00 alone. These results indicate that additional blue light does not affect Φ_PSII_ and Φ_NPQ_ much in green lettuce. On the other hand, red lettuce showed a shift to lower PPFDs in all the blue light treatments with the intersection points of Φ_PSII_ and Φ_NPQ_ occurring at a lower range of 500–800 μmol m^−2^ s^−1^ (Figures 6F–I) in comparison to the Control (950 μmol m^−2^ s^−1^). The additional amount of blue light triggers a decrease in Φ_PSII_ and a concomitant increase in the Φ_NPQ_ and the heat dissipation from PSII (NPQ). These mechanisms are employed by the plants to protect the leaf from possible light-induced damage (Maxwell and Johnson, 2000). Moreover, 1B 06-08 (Figure 6G) again had the strongest effect on Φ_PSII_ illustrating that a predawn (from 06:00 to 08:00) application of blue light might affect the quantum efficiency of PSII more than a post dawn application. It is worth mentioning that the crossing of Φ_PSII_ with Φ_NPQ_ does not represent the whole picture, but it is the crossing of Φ_PSII_ with Φ_NPQ+NO_ that informs at what level the leaf shifts from prioritizing photochemistry to prioritizing heat dissipations, since both Φ_NPQ+NO_ dissipate heat. In conclusion, our results indicate that additional blue LED light applications influence the photosynthetic performance of lettuce. They can be assessed with the chlorophyll fluorescence parameters, which could be used as indicators for possible stressful events.

### Secondary metabolism and pigmentation

In our studies we identified chlorogenic acid, caffeic acid, chicoric acid, cyanidin 3-O-(6″-malonyl-glucoside), quercetin glucuronide, and quercetin malonyl glucoside in both green and red lettuce, though in different amounts (Figures 7, 8). Their amount was higher in all blue LED treatments, although it is not clear which one of the blue light treatments had the most pronounced effect. In more detail for “Lollo Rossa,” we observed that the predawn application 1B 06-08 showed the highest amount of phenolic acids as well as flavonoids, being significantly different from the Control. The 1B 21-08 exhibited a similar trend for the phenolic acids, but not for all the flavonoids. The 2B 17-19 and 1B 17-19 were not always significant from the Control. This highlights the fact that although blue light is involved in the formation of SMs, the latter might be influenced in an independent manner depending on the amount of additional blue light. The effect is more prominent in red leaf lettuce (Lollo Rossa), indicating the cultivar dependence (Figures 7D–F, 8D–F). Since similar results have been found in two cultivars of *Phalaenopsis* that were more or less prone to red colouration during production (Ouzounis et al., 2014b), it also indicates that leaves of green and red coloration have different ecophysiological strategies with respect to acclimation in different spectra. In addition, 1B 06-08 demonstrated the highest amount of all the phenolic compounds, which also had the lowest ETR as mentioned before (Figures 5, 7, 8). This indicates that a blue LED application in the early morning could create a physiological state that induces the accumulation of phenolic compounds. Regarding the mechanism behind the induction of SMs, it has been reported that the activity of phenylalanine ammonia-lyase (PAL), which is a key enzyme in the phenylpropanoid pathway, was stimulated by blue LED irradiation (Heo et al., 2012). In addition, it has been shown that PAL gene expression was activated by monochromatic blue LEDs in lettuce (Son et al., 2012). Consequently, blue light is possibly involved in the activation of the biosynthetic pathway for these phytochemicals.

Plant pigments receive substantial research attention due to their significant involvement in light harvesting activities and stress physiology. Carotenoids are red, orange, or yellow pigments providing protection when plants are over-exposed to light via dissipation of excess energy and free radical detoxification (Lattanzio et al., 2006; Wink, 2010). Moreover, their contribution to photosynthesis is clear through harvesting and transferring light energy to chlorophyll molecules (Davies, 2004; Frank et al., 2004). In particular, chl *a* and *b* molecules are vital pigments, which allow light absorption and transfer of light energy in the reaction centers of the photosystems. Depending on the cultivar, the chlorophyll and carotenoid content varied, but in general all pigments were increased with additional blue light. Indeed, in our study, we identified chl *a*, chl *b*, neoxanthin, violaxanthin, zeaxanthin, lutein, and β-carotene under the supplementary blue LED treatments (Figure 9). In more detail for “Batavia,” chl *a*, chl *b*, and lutein did not differ from the Control, while only 1B 17-19 was different from the Control for violaxanthin and 2B 17-19 and 1B 17-19 was different from the Control for β-carotene, respectively (Figures 9A–G). For “Lollo Rossa,” chl *a* and chl *b* content was significantly different from the Control in 2B 17-19 and 1B 17-19, in contrast with “Batavia,” where no difference observed; although neoxanthin did not show any difference, lutein and violaxanthin showed the same response as the phenolic acids and flavonoids, with the predawn application 1B 06-08 being significantly different from the Control (Figures 9H–N). These findings indicate that the effect of blue supplementary light is cultivar dependent. Blue light is important for chlorophyll formation (chl *a* and chl *b* absorb blue light at approximately 450 and 470 nm), so the additional amount of blue is absorbed and utilized to increase the concentration of chlorophyll (Dougher and Bugbee, 1998; Davies, 2004). Xanthophylls and carotenes are major classes of carotenoids. Particularly, violaxanthin can be epoxidised to zeaxanthin via antheraxanthin under excessive light (Li et al., 2000). Neoxanthin is also an important xanthophyll, being an intermediate in the biosynthesis of abscisic acid (Davies, 2004), which in turn can affect other photosynthetic characteristics, such as stomatal opening and closure (Giday et al., 2014). Lutein and β-carotene are key components in the light-harvesting complexes of leaves. Additionally, it has been reported that leaf senescence and photobleaching occurred under high light intensity in the absence of lutein (Niyogi et al., 1997, 2001). This highlights the importance of these molecules for photoprotection. The chl *a:b* ratio ranged from a 2:1 to 3:1 and varied significantly among the treatments, as also shown on lettuce after exposure to UV (Caldwell and Britz, 2006).

Examples from the literature have also characterized the effect of light on the amount of SMs. In red leaf lettuce, chlorogenic acid, anthocyanin, and total phenol content was increased when exposed to additional blue LED lighting (Johkan et al., 2010). In baby leaf lettuce, the concentration of anthocyanin, phenolics, xanthophylls, and β-carotene was increased under blue irradiation (Li and Kubota, 2009; Samuolienė et al., 2013). However, there are also examples in the literature indicating no significant differences in the amount of phenolic acids, flavonoids, and pigments when plants were exposed under additional blue LED lighting. For instance, no essential difference has been reported in chl *a* and *b* content in *Dieffenbachia amoena* and *Ficus elastica* (Heo et al., 2010) and no significant difference in carotenoids was found in Boston lettuce under LED lighting (Martineau et al., 2012). Therefore, the blue light response is species and cultivar dependent as the production of phenolics and pigments depends on environmental, physiological and biochemical factors (Wink, 2010). Light is one of the most influential factors for regulating secondary metabolism, but more investigation is needed to understand how plants acclimate to different spectral compositions.

We have also shown a positive correlation between the SMs and the quantum efficiency of PSII (Figure 10). The amount and timing of blue lighting affects Φ_PSII_, which correlates well with the amount of phenolic acids, flavonoids, and carotenoids. The two cultivars respond very differently, where the green “Batavia” is unaffected, while the red “Lollo Rossa” shows a clear increase in all three groups of SMs compared to the Control level. However, both cultivars seem to follow the same relationship between Φ_PSII_ and SMs, with “Batavia” having lower concentration of SMs and slightly higher Φ_PSII_ than “Lollo Rossa.” The SMs demonstrate antioxidant, antimicrobial, and photoprotective activities (Kefeli et al., 2003; Wink, 2010), where the latter directly affects the functionality of photosynthesis. When the amount of SMs increased in “Lollo Rossa,” the Φ_PSII_ decreased with a concurrent increase of the Φ_NPQ_ as the light regulated part of the total thermal dissipation (Φ_NPQ+NO_). Our experiment was done under low natural light conditions to prevent photoinhibition. In an ecophysiological context the blue light signals growth in an open environment under clear blue sky where photoprotection against excess light is a good strategy. Our results indicate that it is not solely high light level that triggers photoprotective heat dissipation in the plant, but also the spectral composition of the light itself.

From a research point of view, our findings underline that the effects of blue LED lighting is cultivar dependent and that red colored cultivars show higher sensitivity to light acclimation than green cultivars. The red-colored leaf species can be used as a tool to study the light acclimation in depth as they have the potential to provide information from both a physiological and a chemical aspect with their demonstrated sensitivity under blue light. In commercial greenhouses, the supplemental blue LED lighting could be used for lettuce since essential trading parameters like fresh/dry weight and morphology are not irreversibly or negatively affected. Additionally, increasing blue light could be beneficial as it increased major secondary metabolites that are of significant importance from a health point of view, especially for edible species like lettuce.

### Conflict of interest statement

The review editor, Brian Grout, declares that, despite being affiliated with the same institution as the author, Eva Rosenqvist, the review process was handled objectively. The authors declare that the research was conducted in the absence of any commercial or financial relationships that could be construed as a potential conflict of interest.
